# Fabrication of Poly(ethylene glycol) Capsules via Emulsion Templating Method for Targeted Drug Delivery

**DOI:** 10.3390/polym12051124

**Published:** 2020-05-14

**Authors:** Shuang Yang, Feng Ding, Zhiliang Gao, Jianman Guo, Jiwei Cui, Peiyu Zhang

**Affiliations:** Key Laboratory of Colloid and Interface Chemistry of the Ministry of Education, School of Chemistry and Chemical Engineering, Shandong University, Jinan 250100, China; shuangyang@mail.sdu.edu.cn (S.Y.); dingfeng@mail.sdu.edu.cn (F.D.); gaozl3411@mail.sdu.edu.cn (Z.G.); guojianman17@126.com (J.G.); jwcui@sdu.edu.cn (J.C.)

**Keywords:** emulsion, poly(ethylene glycol), polymer capsules, targeting drug delivery

## Abstract

To reduce nonspecific interactions and circumvent biological barriers, low-fouling material of poly(ethylene glycol) (PEG) is most used for the modification of drug nanocarriers. Herein, we report the fabrication of PEG capsules via the free-radical polymerization of linear PEG or 8-arm-PEG using an emulsion templating method for targeted drug delivery. Doxorubicin (DOX) could be loaded in capsules via electrostatic interactions. The obtained capsules composed of 8-arm-PEG result in a lower cell association (2.2%) compared to those composed of linear PEG (7.3%) and, therefore, demonstrate the stealth property. The functionalization of cyclic peptides containing Arg-Gly-Asp (cRGD) on PEG capsules induce high cell targeting to U87 MG cells. A cell cytotoxicity assay demonstrates the biocompatibility of PEG capsules and high drug delivery efficacy of the targeted capsules. The reported capsules with the stealth and targeting property provide a potential platform for improved drug delivery.

## 1. Introduction

Traditional chemotherapy for cancer treatments could induce side effects because of the nonspecific distribution of drugs into normal tissues. Drug carriers hold great potential to improve drug encapsulation and delivery efficacy [1,2]. The biological barriers could prevent the drug carriers from reaching the tumor site. For example, the drug carriers as foreign substances could be quickly eliminated by phagocytic cells [3]. Therefore, in order to reduce nonspecific interactions with phagocytic cells and prolong the circulation time of the carriers, low-fouling materials have been used to modify the surface of the carriers [4,5,6].

Hydrophilic polymers, such as the mostly used poly(ethylene glycol) (PEG), endow the drug carriers with a low-fouling property because of the formation of a hydration layer on the carrier surfaces [7,8]. PEG-based stealth particles are typically prepared by using self-assembly and surface modification. PEG-containing amphiphilic block copolymers can self-assemble into particles with stealth performance [9]. However, the morphology and size of self-assembled particles are difficult to control, and the preparation of block copolymers usually requires critical synthetic techniques. Modification of PEG on the surface of particles could avoid fast clearance by immune systems [10]. However, the density of PEG modified on the surface of drug carriers could affect their stealth property, which is difficult to control due to steric hindrance [11,12,13]. Instead of PEGylation, PEG particles mainly composed of PEG itself can exhibit prolonged circulation time and avoid nonspecific interaction via tuning the length of the PEG chain [14,15].

PEG particles could improve circulation time and decrease the nonspecific interactions with normal cells, which may also decrease interactions with tumor cells and cellular uptake of drug carriers. It is a challenge to balance the stealth and targeting ability of drug carriers. Therefore, targeting drug carriers have been designed and fabricated by modification of specific groups, such as antibodies, polypeptides, and aptamers [16,17,18]. The cyclic Arg-Gly-Asp (cRGD) peptide is an active targeting ligand, which could specifically bind integrin α_v_β_3_ overexpressed on the surface of cancer cells [19]. Chen et al. reported Au nanoparticles modified with PEG and cRGD, which showed low macrophage cell uptake and high cancer cell targeting ability [20].

Templating methods have been widely used to prepare drug carriers with controllable properties (e.g., size, wall thickness, composition, stability, and surface function) [21]. According to the characteristics of the templates, they could be classified as a hard template and soft template. Hard templates included silica, cuprous oxide, calcium carbonate, polystyrene, and ferroferric oxide [22,23,24,25,26], while soft templates included emulsion drops, bubbles, and proteins. Emulsion droplets as templates are easily prepared by mixing two or more immiscible liquids and can be removed under mild conditions, resulting in the most common soft templates [27]. Small molecular substances can be easily encapsulated and released from hollow particles synthesized using emulsion droplets templating. In recent years, hydrolyzed and concentrated dimethyldiethoxysilane (DMDES) has been used to prepare monodisperse oil-in-water emulsion droplets [28,29,30]. The size of emulsion droplets prepared by this method could be controlled from hundreds of nanometers to several micrometers via tuning the concentration of DMDES, ammonia, and the reaction time [31]. The DMDES emulsion templating method has represented a promising strategy to prepare drug carriers.

**Figure 1:**
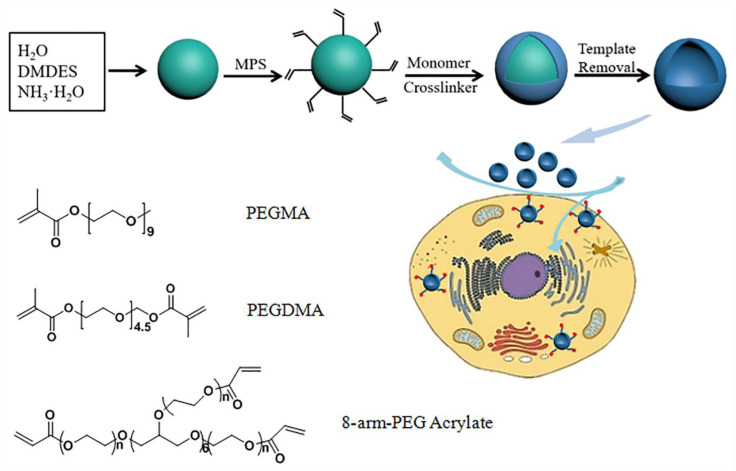
Scheme of the preparation of capsules by using dimethyldiethoxysilane (DMDES) emulsion templating method.

Herein, we report the synthesis of low-fouling capsules based on free radical polymerization on the surface of dimethyldiethoxysilane (DMDES) emulsion droplets (Figure 1). The linear monomer of poly(ethylene glycol) methyl ether acrylate (PEGMA) and poly(ethylene glycol) dimethacrylate (PEGDMA) were used as the building block and cross-linker, respectively, for the fabrication of PEG capsules (LPEG capsules). Further, 2-aminoethyl methacrylate hydrochloride (AEMA) was used as monomers during the preparation of capsules for further modification of the fluorescent dye. A multi-arm monomer of 8-arm-PEG-acrylate (8-arm-PEG-ACLT) as the alternate of PEGMA was also used for preparation of PEG capsules (MPEG capsules). The template could be removed under mild conditions (i.e., ethanol). MPEG capsules showed a better stealth property than LPEG capsules. Modification of the cRGD group on MPEG capsules endowed them with specific targeting properties. In addition, the anti-cancer drug doxorubicin (DOX) was encapsulated into the capsules by electrostatic interactions. We prepared PEG capsules different from PEGylation to increase the PEG density of drug carriers by using the emulsion templating method, where the templates could be removed at mild conditions. The stealth and targeting MPEG capsules showed enhanced cellular uptake and improved drug delivery efficacy. The capsule with the tunable size and stealth property provides a promising way for chemotherapy.

## 2. Materials and Methods

### 2.1. Materials

Dimethyldiethoxysilane (DMDES), 3-(trimethoxysilyl)propyl methacrylate (MPS), poly(ethylene glycol) methyl ether acrylate (PEGMA, *M*_n_ = 480), and 2-(dimethylamino)ethyl methacrylate (DMAEMA) were purchased from Sigma-Aldrich (St. Louis, MO, USA). In addition, 2-aminoethyl methacrylate hydrochloride (AEMA) was supplied by J&K Scientific Co., Ltd., Beijing, China. Poly(ethylene glycol) dimethacrylate (PEGDMA) was obtained from Tokyo Chemical Industry (Tokyo, Japan). Further, 8-arm-PEG-ACLT (20 kDa) was purchased from JenKem Technology Co., Ltd., Beijing, China. Doxorubicin hydrochloride (DOX) was purchased from Aladdin (Shanghai, China). Ammonium hydroxide (NH_4_OH content 28%–30%), sodium dodecyl sulfate (SDS), potassium persulfate (KPS), and ethanol were obtained from Sinopharm Chemical Reagent Co., Ltd., Beijing, China. Dulbecco’s modified Eagle’s medium (DMEM) and Dulbecco’s phosphate-buffered saline (DPBS) were purchased from Neuronbc, Beijing, China. Fetal bovine serum (FBS) and Trypsin were obtained from Gibco, Carlsbad, CA, USA.

### 2.2. Preparation of the DMDES Emulsion Templates

The monodisperse oil-in-water emulsion templates for the fabrication of capsules were prepared by using a previously reported method [29,30]. A typical condition to prepare DMDES emulsion templates with an average size of 450 nm was given as follows: SDS (12 mg) was dissolved in 20 mL of ammonia solution (2% *v/v*). Subsequently, DMDES (400 μL) was added into the solution with a vortex for 1 min. The mixture was stored for 16 h without stirring. Conditions for preparing emulsion templates with other sizes are listed in Table 1. To modify the double bond on the surface of the droplet, MPS (25 μL) was added into the emulsion and stirred at 25 °C for 3 h.

### 2.3. Preparation of the Capsules

PEGMA (50 mg), PEGDMA (5 mg), and AEMA (4 mg) were added into DMDES emulsion (20 mL), followed by stirring for 0.5 h under a nitrogen atmosphere. The initiator of KPS solution (2 mL, 3 mg/mL) was added into the suspension and was continuously stirred for 6 h at 65 °C. Emulsion templates were removed by ethanol (5 mL). The LPEG capsules were separated by centrifugation at 8000 rcf for 5 min and washed with ethanol and water three times, respectively. For the preparation of MPEG capsules, PEGMA was replaced by 8-arm-PEG-ACLT. To label the capsules for cell studies, the capsules in water were incubated with Cy5-NHS (5 μL, 1 mg/mL in DMSO) in the dark.

### 2.4. Preparation of the Targeting Capsules

The thiol-ene reaction, that is, the hydrothiolation of the C=C bond, could proceed quickly under photocatalysis [32]. The targeting cRGD-8-arm-PEG monomer was synthesized by cRGDfC and 8-arm-PEG-ACLT under UV irradiation for 12 h. cRGD-modified MPEG capsules (MPEG-RGD capsules) were fabricated by using the same method for the preparation of MPEG capsules.

### 2.5. Detection of the Stability of Capsules

LPEG capsules and MPEG capsules at a concentration of 1 mg/mL were dispersed in a DMEM medium supplemented with 10% FBS at 37 °C under stirring, respectively. The diameters of the capsules dispersed in aqueous solution at 25 °C were monitored by dynamic light scattering (DLS, Zetasizer Nano ZS90, Malvern Instruments Ltd., Worcestershire, UK) after 0, 4, 8, 12, and 24 h incubation.

### 2.6. Cellular Association

HeLa cells were seeded at a density of 5 × 10^4^ cells/well into 24-well plates and incubated overnight at 37 °C in a 5% CO_2_ incubator. Cy5-labeled LPEG capsules and MPEG capsules with various concentrations (0, 5, 10, 20, and 50 μg/mL) were added into the complete culture medium. After 6 and 24 h incubation, the medium was removed, and the cells were rinsed with DPBS three times, followed by trypsin digestion and resuspension in DPBS. Finally, the fluorescence intensity was measured by a flow cytometer (ACEA, Novo Cyte 3009, San Diego, CA, USA) to analyze the cellular association. The fluorescence signal can be detected by the APC channel of flow cytometry.

### 2.7. Capsules Targeting Analysis

U87 MG and HeLa cell lines were seeded into 24-well plates at a density of 5 × 10^4^ cells/well and allowed to adhere overnight. Subsequently, Cy5-labeled MPEG capsules and MPEG-RGD capsules with various concentrations were added into the culture medium. After 4 h incubation at 37 °C in a 5% CO_2_ incubator, the medium was replaced with fresh medium and rinsed with DPBS three times followed by trypsin digestion for 5 min. The cells were resuspended in DPBS, and the targeting property was analyzed by a flow cytometer.

### 2.8. DOX Loading

DOX, as an anticancer drug model, was loaded into capsules and measured for release. In order to load DOX into the capsules, MPEG capsules or MPEG-RGD capsules (10 mg) in NaOH solution (3 mL, pH 8) were mixed with DOX (3 mg) in the dark. After 24 h stirring at room temperature, the capsules were separated by centrifugation at 8000 rcf for 5 min and washed with water to remove the unloaded DOX.

### 2.9. Cytotoxicity Assessment

Cellular viability was evaluated by U87 MG cell lines after incubation with capsules. U87 MG cells were cultured in DMEM medium supplemented with 10% FBS, 1% penicillin, and streptomycin in the 37 °C, 5% CO_2_ incubator. U87 MG cells were seeded into 96-well plates at a density of 1 × 10^4^ cells/well and allowed to adhere overnight. The culture medium was replaced with fresh culture medium containing DOX@MPEG, DOX@MPEG-RGD capsules, or free DOX, respectively, with various concentrations of DOX for 24 and 48 h at 37 °C. To assess the cytotoxicity of capsules, MPEG and MPEG-RGD capsules were incubated with U87 MG cells for 24 and 48 h, whose concentrations were the same as DOX@MPEG and DOX@MPEG-RGD capsules. Subsequently, MTT (Thiazolyl blue tetrazolium bromide) solution (10 μL, 5 mg/mL) was added to the medium in each well and incubated at 37 °C for 4 h. In the presence of succinate dehydrogenase and cytochrome C, water-insoluble formazan could generate via the reduction of MTT in live cells, which could not generate in dead cells. The medium was removed and DMSO (100 μL) was added to each well to dissolve the formazan salt. The absorbance of formazan in each well was measured at 570 nm by a plate reader (TECAN, Spark 10M, Männedorf, Switzerland) after shaking for 15 min.

### 2.10. Cellular Uptake of Capsules

The cellular uptake and DOX distribution after incubating U87 MG cells with DOX@MPEG capsules and DOX@MPEG-RGD capsules were imaged by confocal laser scanning microscopy (CLSM, Leica, TCS SP8 STED 3X, Weztlar, Germany). Every point in the confocal plane of samples could be scanned via pointolite, which was formed by a laser scanning beam. The images of samples were reconstructed by a photomultiplier tube and displayed on the computer screen. U87 MG cells were seeded into four chambered confocal dishes at a density of 5 × 10^4^ cells/dish and allowed to adhere overnight. Then, DOX@MPEG capsules and DOX@MPEG-RGD capsules (DOX final concentration of 5 μg/mL) were added into confocal dishes. After 4 h incubation at 37 °C in a 5% CO_2_ incubator, the medium was removed. The U87 MG cells were washed with DPBS (500 μL) three times and fixed with 4% paraformaldehyde (500 μL) for 15 min at 37 °C. Then, the cell nuclei and membranes were stained with Hoechst 33342 (5 μg/mL, 500 μL) for 10 min and WGA-AF488 (1 μg/mL, 500 μL) for 6 min, respectively. Finally, the U87 MG cells were imaged by CLSM.

### 2.11. Characterization Methods

Morphologies of capsules were characterized by transmission electron microscopy (TEM, JEOL JEM-1400, Tokyo, Japan). TEM images were obtained using an accelerating voltage of 120 kV. The size distribution and zeta potential were performed by a Zetasizer (Nano ZS90, Malvern Instruments Ltd., Worcestershire, UK) at 25 °C with water as a solvent. Quantification of DOX was analyzed by a UV-vis spectrophotometer (Shimadzu, UV-2600, Kyoto, Japan). The concentration of DOX could be measured by UV-vis measurement at 490 nm. The fluorescent images were obtained by a CLSM (Leica, TCS SP8 STED 3X, Weztlar, Germany). CLSM images were obtained under different fluorescent channels when cells and capsules were stained with different dyes. Cellular uptake was measured by using a flow cytometer (ACEA, NovoCyte 3009, San Diego, CA, USA).

## 3. Results and Discussion

### 3.1. Synthesis and Characterization of Capsules

**Figure 2:**
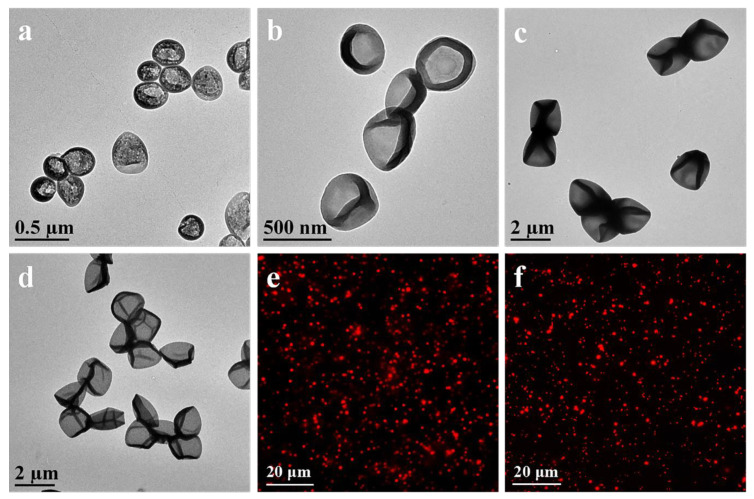
TEM images of the linear monomer of PEG (LPEG) capsules with an average size of (**a**) 230, (**b**) 450, (**c**) 1200, and (**d**) 2000 nm. Fluorescence microscopy images of Cy5-labeled (**e**) LPEG capsules, and (**f**) MPEG capsules with an average size of 1200 nm.

LPEG capsules were prepared via the DMDES emulsion templating method. Compared to a hard template, the soft DMDES template could be removed by using a simple method, such as dissolution in ethanol solution. Monodisperse oil-in-water emulsion droplets could be prepared by the hydrolysis and concentration of DMDES. In this system, the size of the emulsion droplets could be controlled from several hundred nanometers to several micrometers by changing the ratio of DMDES, ammonia, and SDS or reaction time. The capsule size was affected by the size of emulsion droplets, which is difficult to be characterized. Thus, LPEG capsules were used to verify the formation of emulsion droplets. TEM images of LPEG capsules (Figure 2a–d) showed the uniform capsules with an average size from 230 to 2000 nm. The size of the capsules increased along with the increase in the DMDES concentration. For example, the size of LPEG capsules increased from 1200 to 2000 nm along with the increasing DMDES concentration from 1% to 2% at the ammonia concentration of 2% and the reaction time of 24 h. In the presence of surfactant, the capsule size decreased along with the decrease in ammonia concentration, DMDES concentration, and reaction time. For example, the capsule size decreased from 450 to 230 nm when the concentration of ammonia and DMDES was decreased from 2% to 1% at the reaction time of 16 h and the SDS concentration of 0.06 wt.%. The emulsion could be stabilized in the presence of SDS, resulting in the small-sized emulsion template.

To modify low-fouling polymers, MPS with double bonds (C=C) was modified on the surface by a polycondensation reaction under alkaline conditions based on the remaining lots of linear -O^–^ terminated oligomers on emulsion droplets. For fabrication of two kinds of low-fouling capsules, PEGMA or 8-arm-PEG-ACLT was modified on the surface of emulsion droplets via precipitation polymerization and subsequent template removal. The complete and wrinkled shape may be attributed to the evaporation of water (Figure 2a–d). Cy5-labeled capsules were prepared via the formation of amide bonds between Cy5-NHS and AEMA. Fluorescence microscopy images of Cy5-labeled LPEG capsules and MPEG capsules demonstrated the formation of monodisperse capsules with an average size of 1200 nm (Figure 2e,f).

### 3.2. Detection of the Stability of Capsules

**Figure 3:**
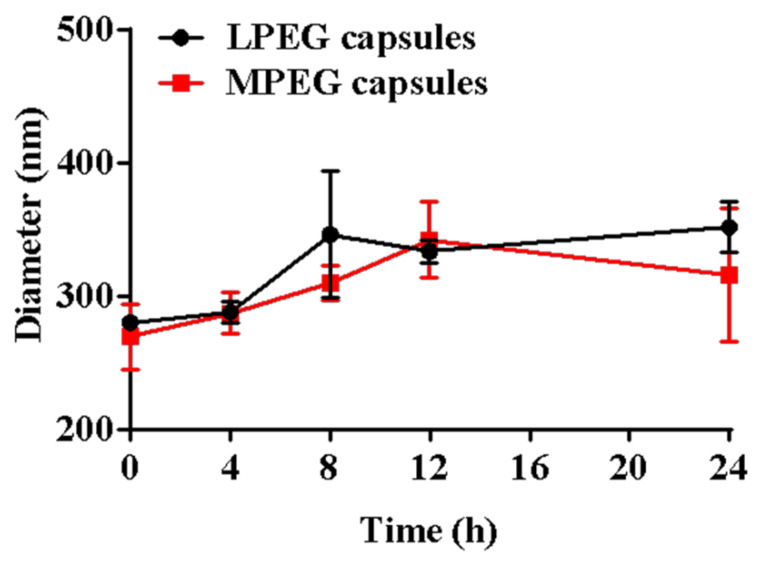
Size change of the capsules in DMEM solution. Error bars represent the standard deviation from three replicate experiments.

To verify the stability of LPEG capsules and MPEG capsules, they were dispersed in DMEM solution to monitor the change in size (Figure 3). The average size of LPEG capsules and MPEG capsules increased by 71.4 and 46.2 nm, respectively, after 24 h incubation, indicating the greater potential of MPEG capsules in resisting protein adsorption. After being injected into the body, MPEG capsules might have a more excellent stability and longer circulation time in the blood compared to LPEG capsules.

### 3.3. Cell Association of Capsules

**Figure 4:**
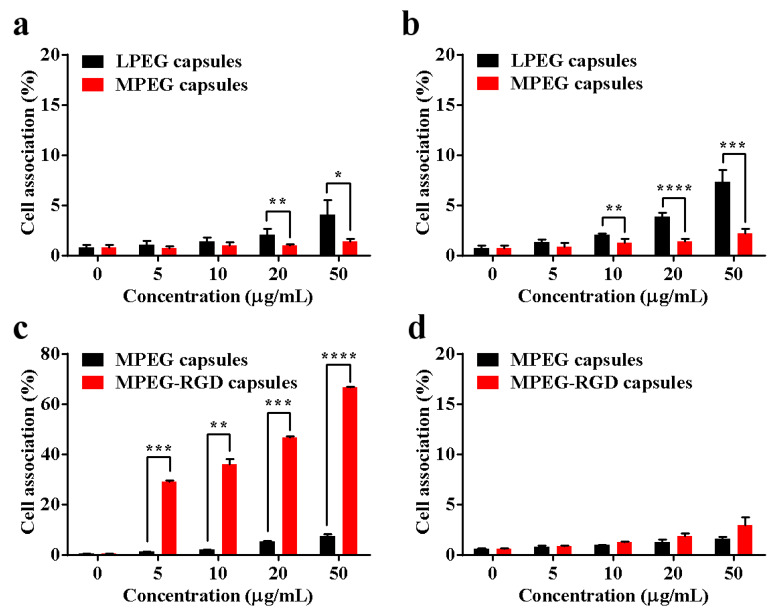
Cell association of Cy5-labeled capsules and HeLa cells for 6 (**a**) and 24 h (**b**). Cell association of Cy5-labeled capsules and U87 MG cells (**c**) and HeLa cells (**d**) for 12 h, respectively (* *p* < 0.05, ** *p* < 0.01, *** *p* < 0.001, and **** *p* < 0.0001). Error bars represent the standard deviation from three replicate experiments.

To verify the stealth property of LPEG capsules and MPEG capsules, cell association including cellular binding and uptake was evaluated after incubation with HeLa cell lines for 6 and 24 h by flow cytometry. As shown in Figure 4a,b, low cell associations of LPEG capsules and MPEG capsules were detected even when the concentration of the two capsules reached 50 μg/mL after incubation for 6 or 24 h with HeLa cells. More importantly, Figure 4b showed the negligible interaction of MPEG capsules and cells after 24 h incubation, indicating excellent antifouling ability of MPEG capsules. As the results show, the antifouling ability of the capsules could be improved along with increasing the chain length and density of PEG. A thick hydrated layer could form on the surface of the MPEG capsules due to the long PEG chains, resulting in the low adsorption of proteins or cells. As a result, the MPEG capsules showed significant differences in cell association compared to LPEG capsules. Therefore, we chose MPEG capsules in the following research.

It should be noted that MPEG capsules could reduce the cellular uptake, which may also decrease the interaction with tumor cells. Thus, we should find a balance between the stealth and targeting performance to avoid recognition of the capsules by the immune system while specifically targeting the diseased tissue. In order to prepare targeting capsules (MPEG-RGD capsules), cRGD was modified to 8-arm-PEG-ACLT before polymerization. After 12 h incubation with U87 MG cells, cell association was analyzed by flow cytometry. As shown in Figure 4c, the cell association of MPEG-RGD capsules was much higher compared to MPEG capsules, which could be attributed to the specific targeting ability of cRGD to the overexpression of the α_v_β_3_ protein on the U87 MG cell surface. For example, the association of MPEG-RGD capsules and U87 MG cells was about 65% after 12 h incubation. There are significant differences (*p* < 0.0001) of cell association between MPEG capsules and MPEG-RGD capsules, which showed a good targeting property. To evaluate the stealth property of MPEG-RGD capsules, they were incubated with HeLa cells. The cell association of MPEG-RGD capsules was negligible, indicating the low influence of cRGD modification on the low-fouling property (Figure 4d).

### 3.4. DOX Loading

**Figure 5:**
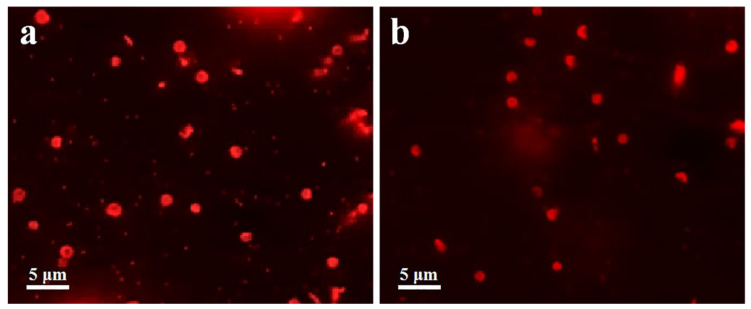
Fluorescence microscopy images of (**a**) doxorubicin (DOX)@MPEG capsules and (**b**) DOX@MPEG-RGD capsules.

**Figure 6:**
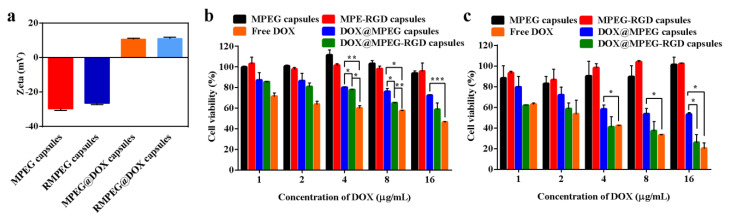
(**a**) Zeta potential of LPEG, MPEG, MPEG-RGD, DOX@MPEG, and DOX@MPEG-RGD capsules. Cell viability of four kinds of capsules to U87 MG cells after 24 h (**b**) and 48 h (**c**) incubation (* *p* < 0.05, ** *p* < 0.01, and *** *p* < 0.001). Error bars represent the standard deviation from three replicate experiments.

DOX, as a common anti-cancer drug, can inhibit the synthesis of RNA and DNA, resulting in the apoptosis of tumor cells [33]. Dox-loaded capsules were prepared by electrostatic interaction. The inverted fluorescence microscopy images based on autofluorescence of DOX showed the well-dispersed DOX@MPEG capsules (Figure 5a) and DOX@MPEG-RGD capsules (Figure 5b) in water. The zeta-potential (Figure 6a) of MPEG capsules and MPEG-RGD capsules were negative due to the presence of silicon oxide in the inner layer of the capsules. The zeta potentials of the two kinds of capsules reversed from negative charge to positive charge due to the loading of DOX [34]. The amount of DOX loaded on the capsules was measured by the absorption at 490 nm. The DOX encapsulation efficiencies of MPEG capsules and MPEG-RGD capsules were calculated to be 61% and 56%, respectively, and the loading efficacies of DOX were 18% and 17%, respectively.

### 3.5. In Vitro Cytotoxicity Assessment of Capsules

**Figure 7:**
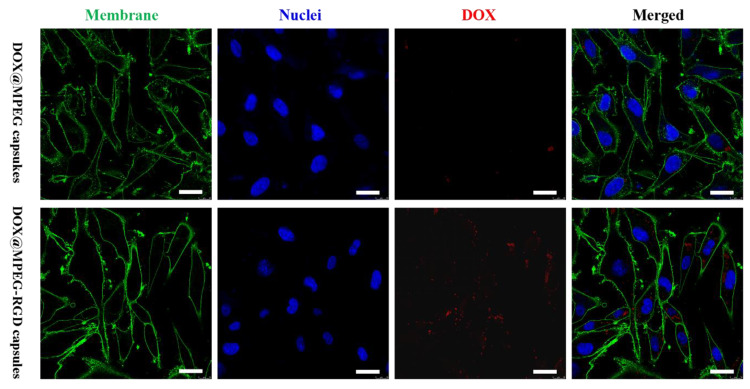
Confocal laser scanning microscopy (CLSM) images of U87 MG cells after co-culture with DOX@MPEG capsules and DOX@MPEG-RGD capsules. Cell membranes were stained with WGA-AF488 (green) and cell nuclei were stained with Hoechst 33342 (blue). Red fluorescence was from the DOX. Scale bars are 50 μm.

To evaluate the cytotoxicity of the capsules, an MTT assay was performed using U87 MG cells. As shown in Figure 6b,c, the cell viability of MPEG capsules and MPEG-RGD capsules at 24 or 48 h was above 90% even when the concentration of capsules was 72 and 79 μg/mL (the corresponding concentration of DOX in DOX@MPEG capsules and DOX@MPEG-RGD is 16 μg/mL), indicating the biocompatibility of the capsules. The cell viability decreased when DOX was loaded into the capsules, which may be caused by the interaction between the positively charged capsules and negatively charged cell membranes. When the concentration of DOX reached 16 μg/mL, the cell viability of DOX@MPEG-RGD capsules was 26% after co-culture with U87 MG cells for 48 h, while the cell viability of DOX@MPEG capsules was 53%. There are significant differences (*p* < 0.05) in cell viability between DOX@MPEG capsules and DOX@MPEG-RGD capsules. The latter showed a higher cell cytotoxicity compared to DOX@MPEG capsules due to the higher cell association of cRGD-modified capsules. The IC_50_ value of DOX@MPEG-RGD capsules (2.72 μg/mL) was similar to that of free DOX (2.42 μg/mL). DOX@MPEG-RGD capsules had a high therapeutic efficiency and low side effects. From CLSM results (Figure 7), the targeted capsules achieved a higher cellular uptake than the non-targeted capsules, which was consistent with the cell cytotoxicity results.

## 4. Conclusions

In this work, LPEG capsules and MPEG capsules were successfully fabricated via free-radical polymerization by the emulsion templating method. The emulsion templates could be removed under mild conditions. The size of capsules was controlled from a hundred nanometers to several micrometers. Both LPEG capsules and MPEG capsules showed good biocompatibility, stability, and low cell association. MPEG-RGD capsules could specifically target U87 MG cells, which overexpressed α_v_β_3_ integrin on the cell membrane surface while maintaining the stealth property against other cells (e.g., HeLa cells) without expression of α_v_β_3_ integrin. In addition, DOX@MPEG-RGD capsules could significantly improve their cell cytotoxicity of U87 MG cells compared to DOX@MPEG capsules. The reported capsules provide a new avenue to fabricate targeted carriers for drug delivery.

## Figures and Tables

**Table 1:** List of parameters for preparation of emulsion templates with different sizes.

Diameter of Emulsion Template (nm)	SDS Concentration (mg/mL)	NH_3_ Concentration (*v/v*)	DMDES Concentration (*v/v*)	Time (h)
2000	0	2%	2%	24
1200	0	2%	1%	24
450	0.6	2%	2%	16
230	0.6	1%	1%	16
